# Density Measurements of Molten LiF–BeF_2_ and LiF–BeF_2_–LaF_3_ Salt Mixtures by Neutron Radiography

**DOI:** 10.1021/acsomega.4c01446

**Published:** 2024-06-10

**Authors:** Jisue Moon, Joanna McFarlane, Hunter B. Andrews, Kevin R. Robb, Molly Ross, Dino Sulejmanovic, Yuxuan Zhang, Erik Stringfellow, Can Agca, Juliano Schorne-Pinto, Theodore M. Besmann

**Affiliations:** † Oak Ridge National Laboratory, Oak Ridge, Tennessee 37831, United States; ‡ University of South Carolina, Columbia, South Carolina 29208-0001, United States

## Abstract

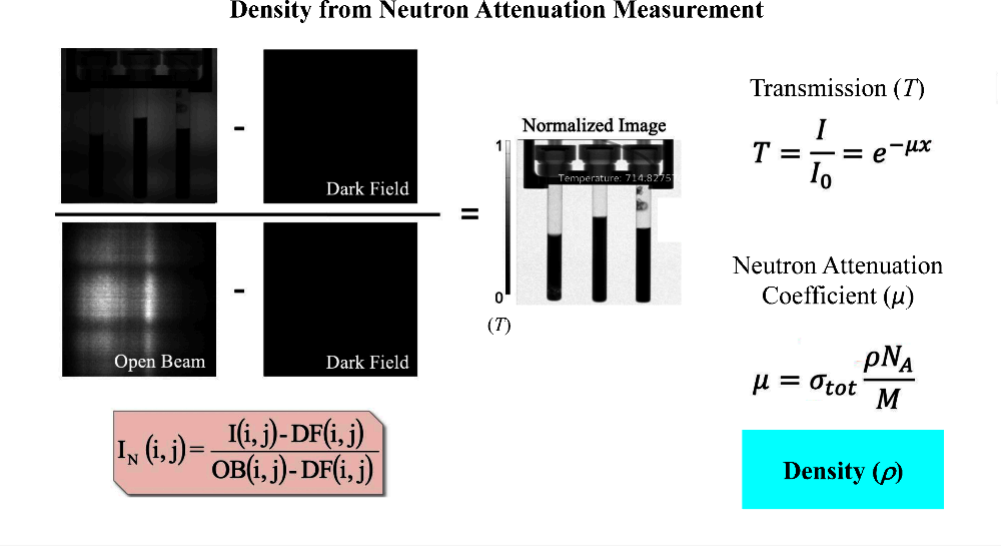

The densities of eutectic (LiF)_2_–BeF_2_ and mixtures of this salt (FLiBe) with LaF_3_ were measured by dilatometry and by neutron attenuation from 673 K to 1,073 K. Because LaF_3_ has a limited solubility in FLiBe, it was necessary to determine the amount of LaF_3_ in solution before the density could be determined. The FLiBe density determination was favorably benchmarked against the literature data. A simple comparison was not available for the LaF_3_–FLiBe mixtures, so extrapolation of published data was necessary based on analysis using the Molten Salt Thermal Properties Database-Thermochemistry, or MSTDB-TC, developed by the US Department of Energy. Solubilities for LaF_3_ in FLiBe ranged from 1 to 4 mol % over 673 to 1,073 K. The salt system was heated and cooled over 24 h to evaluate potential changes in composition and hysteresis during the measurement. Changes in the meniscus were observed, and these were included in the correction for density determinations. Salt surface tension may have led to supersaturation of LaF_3_ in the salt because the solubility curve was nonlinear with respect to the inverse temperature, as would be expected for an ideal system. Surface tension measurements are currently underway to test this hypothesis.

## 1 Introduction

Molten halide salts are being considered for fission and fusion applications. For fission, molten salt reactors (MSRs) provide the following advantages: a flexible fuel cycle instead of ceramic fuel used in light-water reactors, operation at close-to-ambient pressure, high-temperature operation for improved thermodynamic efficiency, and improved reactor safety by avoiding a potential steam-Zircaloy reaction and H_2_ generation. For fusion, molten salts are considered as blanket materials to produce tritium for fuel. Because of the wide range of reactor designs and salt compositions, fuel salts for MSRs are an active area of research into their thermochemical and thermophysical properties.

In particular, because of its neutronic properties, a eutectic mixture of 2LiF–BeF_2_ (FLiBe) salt is a leading candidate for a diluent carrier salt and coolant salt. Historically, fluoride salts were investigated extensively at Oak Ridge National Laboratory (ORNL) for the Aircraft Reactor Experiment (ARE) that used a mixture of NaF–ZrF_4_–UF_4_^1^ and for the Molten Salt Reactor Experiment (MSRE) that was powered up with a mixture of LiF–BeF_2_–ZrF_4_–UF_4_.^2^ Globally, MSRs development is growing rapidly.^3^ Some examples of MSRs that use FLiBe in the US include Abilene Christian University (ACU) and Kairos Power that are building demonstration MSRs.^4,5^ ACU plans to use a salt mixture similar to MSRE,^4^ whereas Kairos plans to use a solid ceramic fuel.^5^ Commonwealth Fusion and XCimer are considering using FLiBe in their fusion designs.^6,7^ Several other developers are designing chloride salt reactors;^8^ notably the MCRE reactor at INL.^9^

The operation of MSRs is affected by the reactors’ thermophysical properties^10^ because they govern the flow of mass and heat through the primary circuit during operation. The properties of most interest include density, heat capacity, thermal conductivity, and viscosity. Optical properties (e.g., emissivity) and interfacial interactions (e.g., surface tension and wettability) are also important. In this work, the focus is on measuring density, because it has wide-ranging effects on system mass, neutronics, and thermal-hydraulic performance. Because of its historic use and current interest, FLiBe is among the more rigorously studied salts. A recent paper summarized the available literature for density,^11^ which mostly has relied on the Archimedes method. Neutron radiography has been used to measure the density of chloride molten salts by dilatometry.^12,13^ Because neutron imaging allows direct observation of the heated salt, it also provides a phenomenological understanding of salt behavior, allowing measurement of contact angle and directly observing hysteresis effects as the salt is thermally cycled.

Thermochemical properties govern the behavior of the components in the salt, including the fuel, fission, and activation products. Understanding the solubility of lanthanides, which are byproducts of the fission reaction occurring in the MSR core, is important, because they make up a significant fraction of the salt-soluble fission products. These fission products affect neutronics and system operation through parasitic absorption of neutrons.^14^ The higher solubility of lanthanides relative to that of actinides in chloride molten salts can be used to process spent nuclear fuel, as demonstrated in the chloride salt electrorefining process at Idaho National Laboratory.^15^

Previously, solubilities of LaF_3_, CeF_3_, and SmF_3_ in LiF–BeF_2_–UF_4_ (62.8–36.4–0.8 mol %) salt as well as YF_3_ and CeF_3_ in NaF–BeF_2_ (61–39 mol %) salt were measured to support MSRE.^16^ Solubilities are also published for ARE salts, including the measurements of BaF_2_, LaF_3_, CeF_3_, SmF_3_, and YF_3_ in NaF–ZrF_4_–UF_4_ (50–46–4 mol %) salt.^17^ Ward et al. extracted samples for analysis from equilibrated (1 h) molten salt mixtures by drawing the sample into a capillary tube through a sintered nickel filter. Accurate analyses for lanthanides in the presence of Na, Zr, and U required a radioactive tracer method, because direct chemical methods gave poor results. Although successful, generation and handling of activated material, some with relatively short half-lives, posed challenges for in situ sampling and handing. Naumov et al. measured the solubilities of LaF_3_, CeF_3_, CeF_4_, NdF_3_, and PuF_3_ in LiF-BeF_2_–ZrF_4_ (65–29–5 mol %).^18^ In their technique, a molten salt was sampled in situ using quartz tubes, and the chemical analysis was conducted by X-ray analysis. Although the lanthanides are generally considered to be salt soluble, their solubilities appear to be limited to a few mol % in fluoride salts.^19,20^

The temperatures of interest in MSRs span from the FLiBe salt’s freezing point (732.2 K^21^) to temperatures approaching 1,100 K. FLiBe systems are commonly designed to operate at 900 K to 1,000 K. The highest temperatures may be encountered during off-normal conditions.^22^

There are several complications for measuring the temperature-dependent solubility of species in molten halide salts. First, salts must be handled under inert conditions because they readily absorb oxygen and moisture. Second, the high temperatures pose a challenge with respect to experimental equipment, handling, and data interpretation (e.g., consideration of thermal expansion in measurement equipment). During freezing, species can migrate, resulting in concentration heterogeneity. To mitigate this effect, measurements can require long periods on the order of hours, to stabilize and prevent phase segregation. Third, for beryllium-containing salts (e.g., FLiBe), special attention is required in salt handling because of the unique health hazards and low permissible exposure limits.

In this study, the mixtures of FLiBe and LaF_3_ were studied to determine the density, which has not been reported elsewhere. This measurement also required determination of the solubility of LaF_3_ in FLiBe. The technique used tracked the mixture through several heating and cooling cycles by neutron radiography. During this process, it became evident that because of the limited solubility of LaF_3_ in FLiBe, the methodology had to be expanded beyond dilatometry, which has been used previously.^12,13^ Hence, the solubility of LaF_3_ in FLiBe was determined using neutron attenuation and was compared to literature values. Once determined, the density of the mixture was reported. The methods used, associated uncertainties, and resulting measurements are discussed herein.

## 2 Experimental Method

FLiBe and mixtures of LaF_3_ and FLiBe in the ratios listed in Table 1 were prepared in an inert glovebox in which moisture and oxygen levels were controlled (e.g., <5 ppm of O_2_, <1 ppm of H_2_O). The FLiBe salt was purified by hydrofluorination,^23^ fused, and crushed into small pieces before being loaded into vanadium sample cans for neutron radiography.^24^ The lithium in the FLiBe salt was of natural isotopic abundance, 7.5% Li-6 and 92.5% Li-7. The as-received anhydrous LaF_3_ (99.9 wt %, Lot X28H013) from Alfa Aesar, now Thermo Fisher Chemicals, was loaded into the cans in small pieces of the desired weight ratios. The inner diameter and thickness of the vanadium cans were 6.040 ± 0.0018 and 0.35 mm, respectively, and 5 cm long, giving a total internal volume of 1.4 cm^3^. Vanadium was chosen for sample containment as it has very little interaction with the cold neutron flux (0.8 < λ < 6.0 Å). The cans were capped with threaded titanium lids under 1 bar argon and sealed with a graphite gasket cut from a 0.8 mm thick sheet. The loaded and sealed samples were not premelted before the introduction into the beamline furnace. Best practice does have a preheating step to remove entrained gas bubbles and voids;^13^ however, a furnace approved for beryllium work was not available off-beam. Thus, the initial heating in the beamline gave rise to voids in the salt mixture that settled only with thermal cycling of the salt.

**Table 1:** Samples Used for Density and Solubility Measurements of LaF_3_ with Neutron Imaging, along with the Mass and Composition Information

	Sample	FLiBe weight (±0.01 g)	LaF_3_ weight (±0.01 g)	Composition (mol % [±1%])
1	FLiBe	2.24	–	66.6% LiF, 33.3% BeF_2_ (100% FLiBe)
2	10% LaF_3_ in FLiBe	2.02	0.22	90.7% FLiBe, 4.3% LaF_3_
3	20% LaF_3_ in FLiBe	1.79	0.45	89.1% FLiBe, 9.3% LaF_3_

Density measurements were conducted using the Multimodal Advanced Radiography Station (MARS) CG-1D beamline of ORNL’s High Flux Isotope Reactor (HFIR). This neutron imaging instrument is situated at the cold neutron guide, and the flight path from the aperture to detector is 6.59 m.^25^ The neutron attenuation of the sample is determined by its mass, path length, and isotopic abundance, from which the density can be determined. Attenuation can be a result of neutron capture or scattering, the former leading to activation and the latter dominating the interaction of neutrons with light elements, such as hydrogen. In these analyses, it is assumed that the attenuation follows the Beer–Lambert law.^26^ Details of the analysis are presented in the Results.

The assembly inserted into the vacuum furnace at the beamline held three sample tubes. After the samples were loaded onto a spacer and the spacer was secured onto the sample assembly, two thermocouples were secured adjacent to the assembly. One was located at the side of the assembly to monitor the sample temperature, and another mounted just above the assembly was used to control the furnace heating elements. These thermocouples enabled the temperature to be maintained within ±5 K of the set temperature inside of the furnace. The assembly was then placed into a vacuum furnace capable of reaching temperatures up to 1,873 K, which is well above the temperatures in these experiments. Before the samples were loaded into the vacuum furnace, it was flushed with ultrahigh-purity nitrogen to remove oxygen and moisture. The furnace was then evacuated, and the samples were slowly heated from 673 K to 1,073 K in increments of 100 K with a heating rate of 0.6 K min^–1^. The samples were held for 30 min at each 100 K interval. The cooling process was also carried out at a rate of 0.6 K min^–1^, with 30 min hold periods at decrements of 100 K. After an initial rapid melt, there were two heating and cooling cycles, during which radiographs were recorded every minute. The uncertainty in the temperature measurement has been estimated as ±5 K, as determined by the difference in readings between two thermocouples located close to the sample.

The mass of each sample was measured during preparation to an uncertainty of ±10 mg, allowing for the determination of its density from the volume of salt obtained through neutron imaging. The resulting neutron images were normalized using open-beam and dark-field images and normalized into transmission images.^13^ The profile of each sample was carefully measured to determine the bottom of the meniscus of the salt and the interface between the vanadium can and the salt. The thermal expansion of the V can was estimated to determine the diameter at each temperature,^27^ which was used to find the conversion from pixels to cm. A description of the full density calculation and error can be found in Supporting Information.

## 3 Results

**Figure 1:**
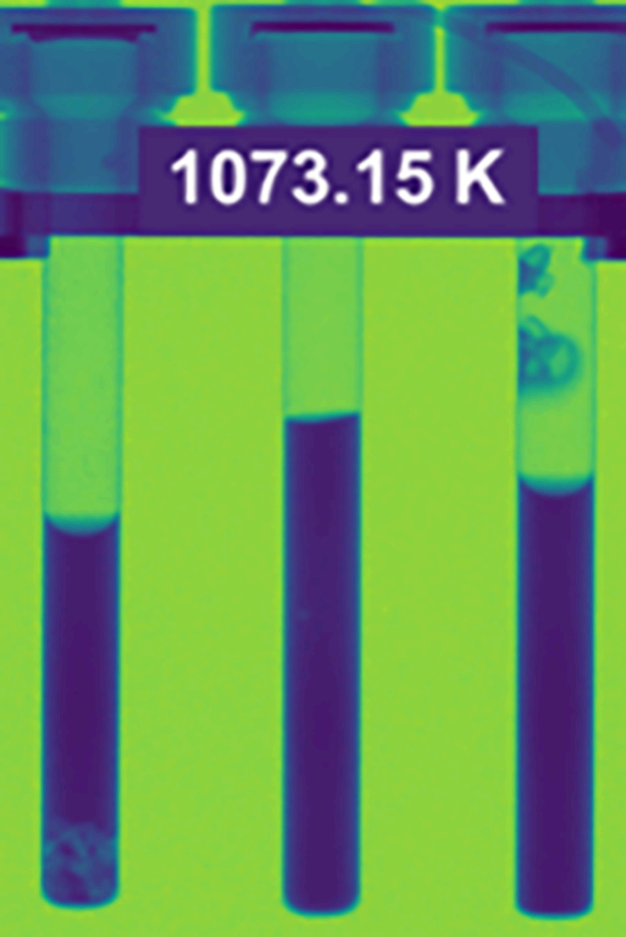
Radiographs of FliBe and LaF_3_ mixtures as inserted perpendicular to the neutron beam: (left) 20% LaF_3_ + FliBe or mixture 2 (middle), FLiBe, and (right) 10% LaF_3_ + FLiBe or mixture 1.

**Figure 2:**
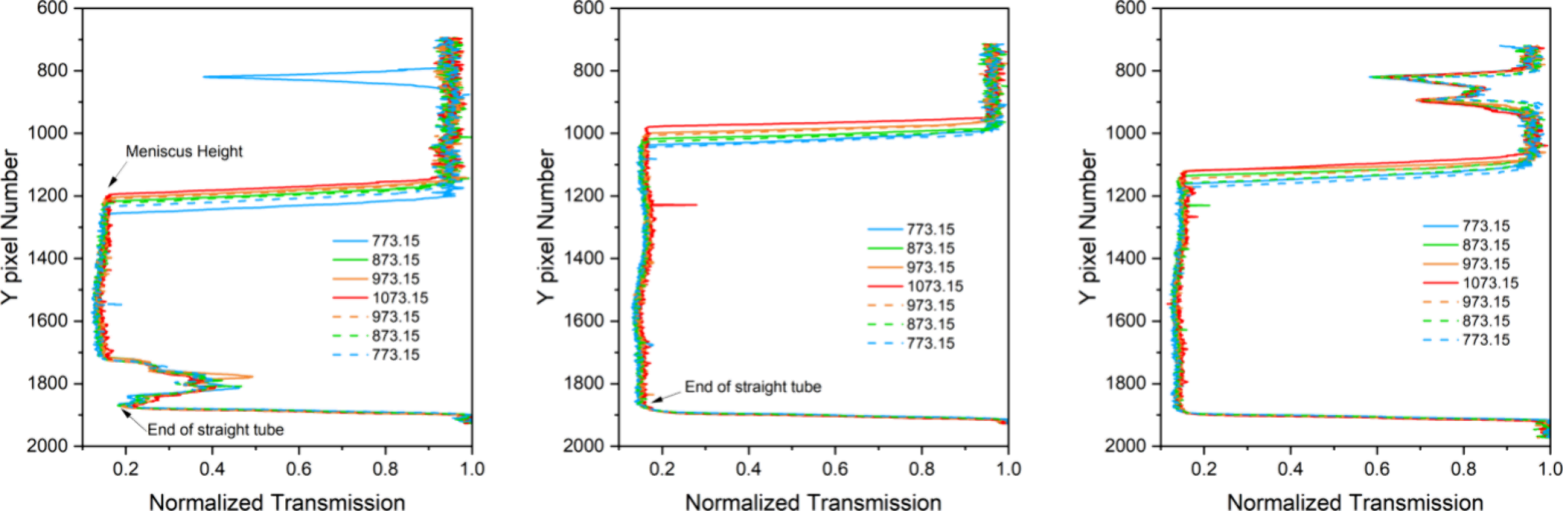
Normalized neutron transmission profile with Y pixel number that is proportional to the vertical distance along the sample can (1 pixel ∼60 μm) for the samples shown in Figure 1: (left) 20% LaF_3_ + FLiBe or mixture 2, (middle) FLiBe, and (right) 10% LaF_3_ + FLiBe or mixture 1. The bottom of the tube is indicated at the lowest Y pixel, where the transmission of the neutrons drops quickly. The bottom of the meniscus is at the Y pixel, where the transmission increases dramatically. The solid and dashed lines represent data collected during heating and cooling, respectively.

Three samples were heated simultaneously in the beam. Figure 1 shows a false color neutron radiograph image taken at 1,073 K. A set of radiographs were collected during the heating and cooling processes, respectively, in increments of 100 K from 773 to 1,073 K, provided in the Supporting Information and shown in Figures S1(a) and (d), and from 1,073 to 773 K, shown in Figure S1 (e)–(g). In the radiograph displayed in Figure 1, the samples were loaded with 10 wt % LaF_3_ (right) and 20 wt % LaF_3_ (left) in FLiBe, subsequently also referred to as mixture 1 and mixture 2, respectively. Neat FLiBe was measured as a reference (middle). Figure 2 presents the transmission profile at each temperature for each sample and shows an increase in height with temperature, as expected. The profiles are based on the entire width of the sample, with the edge pixels in the radial direction measured in the empty tube above the salt. Note that the radiographs that contributed to Figure 2 are shown in Figure S1.

Salt densities were determined in two different ways: by volume change (i.e., dilatometry) and by measurement of changes in neutron attenuation. The next two sections discuss how these were applied to the FLiBe salt on its own, as its density was used as a reference for measurements on the FLiBe–LaF_3_ salt mixtures.

### 3.1 Density Measurement of FLiBe Using Volume Change with a Known Mass

**Figure 3:**
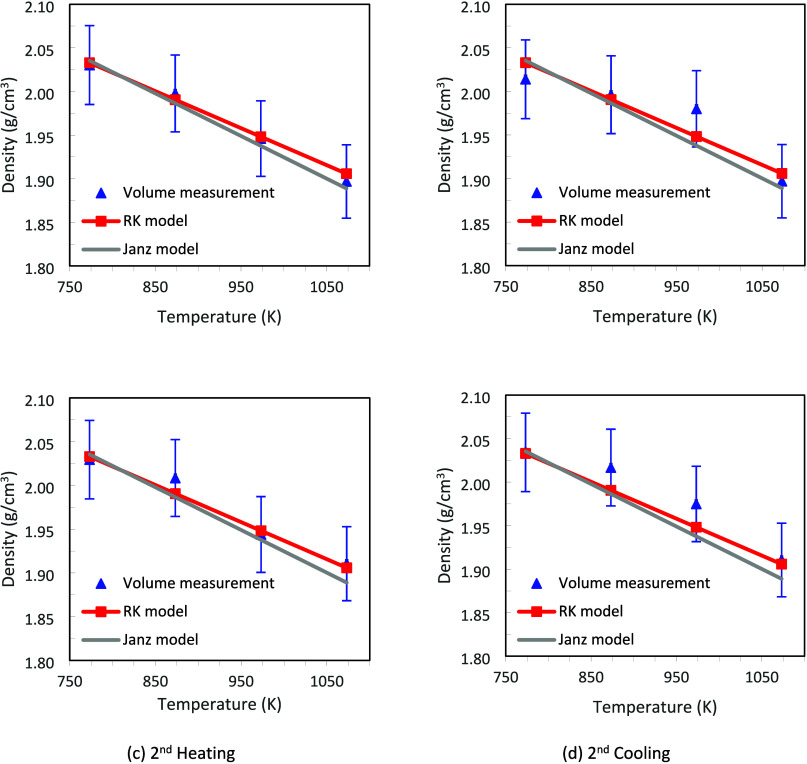
FLiBe density as a function of temperature calculated from estimated volume in image, Janz model,^28^ and Redlich–Kister (RK) model.^29^

**Figure 4:**
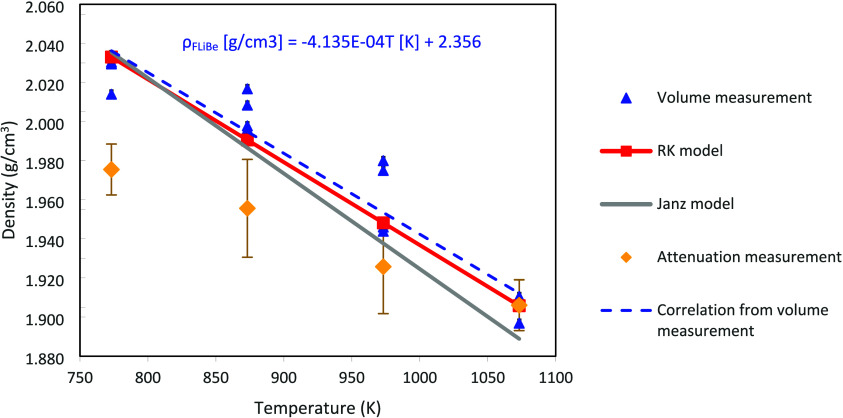
Density comparison between volume measurement and the attenuation coefficient for FLiBe.

The FLiBe sample showed a relatively consistent transmission profile during the heating and cooling processes, as shown in Figure S2, and the density of the FLiBe was calculated every 100 K from 773 to 1,073 K. Radiographs were recorded as the salt went through two consecutive heating and cooling steps, and the density at each 100 K increment or decrement was recorded and compared with the reference. Because the salt was powder when introduced into the canister, the salt had to be heated to melt and become homogenized before density data could be collected. Once homogenization was achieved, radiographs were collected during slow cooling and heating. Figure 3 compares the measured density of FLiBe during sequential heating and cooling steps to the correlation reported by Janz,^28^ and those calculated using the Redlich–Kister (RK) method that includes binary interactions.^29^ Error bars, approximately ±2.2%, for the density measurements are provided which were calculated using the method presented in the Supporting Information. The measured density values during the heating cycle match the RK model. The density values during the cooling cycles are also still within the margin of error for the RK model but deviate further from the reported values. This is most likely due to the larger and potentially nonuniform meniscus as the sample cools, which may not be captured accurately in the volume of the curved surface. A correlation for density based on a linear fitting of all heating a cooling data points is presented in Figure 4.

### 3.2 Density of FLiBe by Neutron Attenuation

The sample containing 10 wt % LaF_3_ or mixture 1 (right sample in Figure 1) showed salt adhesion to the wall of the vanadium vessel during the initial heating. These crystals did not dissolve back into the melt and stayed at the same position at 1,073 K even after repeated cycling. Although most of the melt under the meniscus showed a relatively uniform transmission in Figure 2(c), the crystal above the melt is likely to be LaF_3_ with a melting point of 1,766 K,^30^ which is well above the maximum temperature of the experiment (i.e., 1,073 K). Because the salt volume is not contiguous for this sample, the density cannot be calculated from the change in the volume of the melt. Furthermore, for the sample with 20 wt % LaF_3_ or mixture 2, the undissolved crystals at the bottom of the melt are evidence that all of the LaF_3_ was not dissolved in the FLiBe melt. The undissolved amount of LaF_3_ was significant, and the height of crystallized LaF_3_ did not change much during the heating and cooling processes. Thus, density measurements through macroscopic changes in volume were not possible for the samples containing LaF_3_.

Because LaF_3_ has poor solubility, as indicated by the observation of undissolved crystals, the density of the mixture could not be calculated from volume change. Alternatively, the density was calculated using an attenuation coefficient. From the attenuation law, the intensity of the neutron beam behind the sample can be calculated following the Beer–Lambert Law, as shown in eq 1
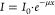
1where *I*_0_ is the incident intensity of the neutron beam, *I* is the weakened intensity of the neutron beam, μ is the attenuation coefficient (cm^–1^), and *x* is the sample thickness (cm). The attenuation coefficient is determined by nuclear density *N* and interaction probability σ for the respective elements constituting the sample. The effective attenuation coefficient μ_eff_ consists of the attenuation coefficients of the involved elements and can thus also be determined as follows
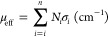
2where *N* is the nuclear density (nuclei·cm^3^) and σ is the microscopic cross section interaction probability of each nuclide with the neutron (cm^2^) beam.

The atomic density (atoms·cm^–3^) is
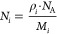
3where ρ is the density (g·cm^–3^), *N*_A_ is the Avogadro constant (atoms·mol^–1^), and *M* is the atomic weight (g·mol^–1^).

If the elemental composition and the cross section of the incident neutron with the elements present in the material examined are known, the density of a sample can be determined by
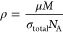
4The radiography images were corrected for variations in the detector current by subtracting the dark image and for inhomogeneities in the beam by subtracting the empty cell radiograph. For neutron attenuation measurements, the images needed to be corrected for the cylindrical geometry of the cell, to determine the thickness of the sample at each pixel. The geometric conversion corrected for changes in path length and for wall thickness that affected path length, *x*, as given in eq 1. The theoretical attenuation coefficients of the isotopes involved were calculated from the iNEUIT database.^31^ The FLiBe density was calculated and compared with the volume measurement and the Janz and RK models in Figure 4.

Using neutron attenuation is a valid approach when all of the major artifacts can be controlled. The major ones are usually (1) beam hardening and (2) scattering background. Beam hardening can become an issue, particularly when different mixtures have very different transmission spectra. In the case of these samples, the transmission is already quite low, and densities are being calculated based on relative changes in this low range. Thus, the beam should be well hardened, and the hardening effect should be equivalent for the different temperatures and samples. Scattering versus absorption can lead to deviations for quantitative imaging. However, as these samples should show similar scattering behavior, the relative change in measured density is expected to be minimal. Additionally, such an effect is minimized with increased sample-to-detector distance, which in this case was approximately 0.5 m. It can be seen that the density by attenuation measurement is lower than the volumetric measurements at high temperatures, being also subject to thermal expansion of the V can. However, as volumetric determinations cannot address systems that have secondary phases or unknown system composition, neutron attenuation was used for the LaF_3_ system as described in the next sections.

### 3.3 Solubility of LaF_3_ in FLiBe from Neutron Attenuation

**Figure 5:**
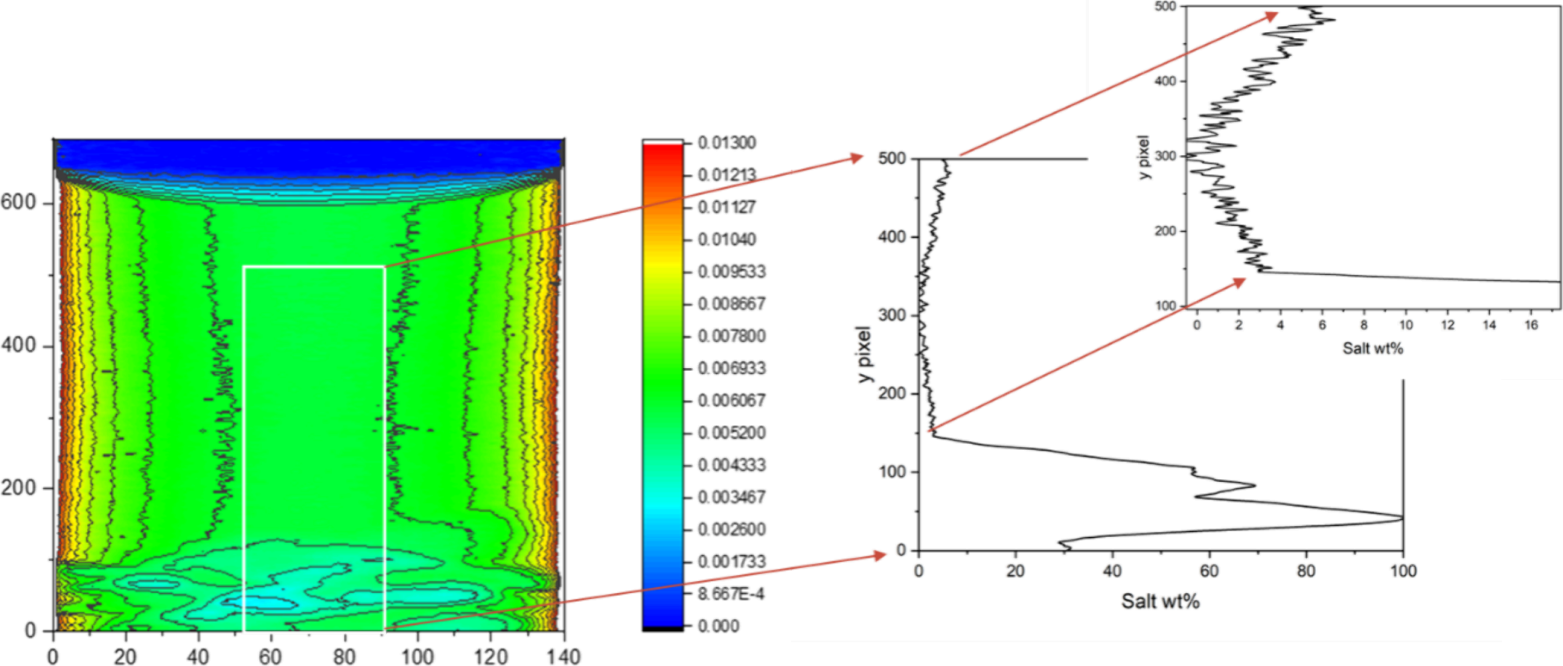
Demonstration of the derivation of attenuation coefficients from neutron radiography data.

The solubility of LaF_3_ in the salt was determined before a density calculation was performed for the LaF_3_–FLiBe mixtures. The compound LaF_3_ has shown poor solubility (less than 4 mol %) in FLiBe salt with additional fluoride materials (i.e., UF_4_, ZrF_4_, and NaF).^17^ The attenuation coefficient of pure LaF_3_ with known density (5.9 g·cm^–3^) and molecular weight (195.9 g·mol^–1^) is 0.6271 cm^–1^, and the measured attenuation coefficient of FLiBe is ∼2.8 cm^–1^ with a variation of 0.02 cm^–1^ between 773 K and 1,073 K.^24^ Because the attenuation coefficient of LaF_3_ is significantly less than that of FLiBe, the solubility of LaF_3_ could be determined from the difference of the attenuation in the mixture relative to pure FLiBe, as diagrammed in Figure 5. The average attenuation was calculated across the highlighted rectangle or the region of interest shown in the figure, avoiding issues related to the cylindrical geometry of the vessel. The attenuation as a function of pixel height was averaged over 40 pixels and is shown as the rectangular region in graph on the left-hand side of Figure 5. The attenuation was averaged along the vertical axis of the sample and converted to a weight percentage. The analysis started above the location of the precipitated solids at the bottom of the graph that correspond to deposited LaF_3_. Solubilities are listed in Table 2.

**Table 2:** Solubility Determinations for Two Mixtures of LaF_3_ in FLiBe between 773 K and 1,073 K

Temp (K)	10000/*T*	Solubility (mol %) in FLiBe + 10 wt % added (LaF_3_) Mixture 1	Solubility (mol %) in FLiBe + 20 wt % added (LaF_3_) Mixture 2
773	12.9	0.77 ± 0.21	0.98 ± 0.13
873	11.5	1.68 ± 0.08	1.87 ± 0.08
973	10.3	2.20 ± 0.60	2.57 ± 0.33
1073	9.3	4.13 ± 0.47	3.96 ± 0.27

**Figure 6:**
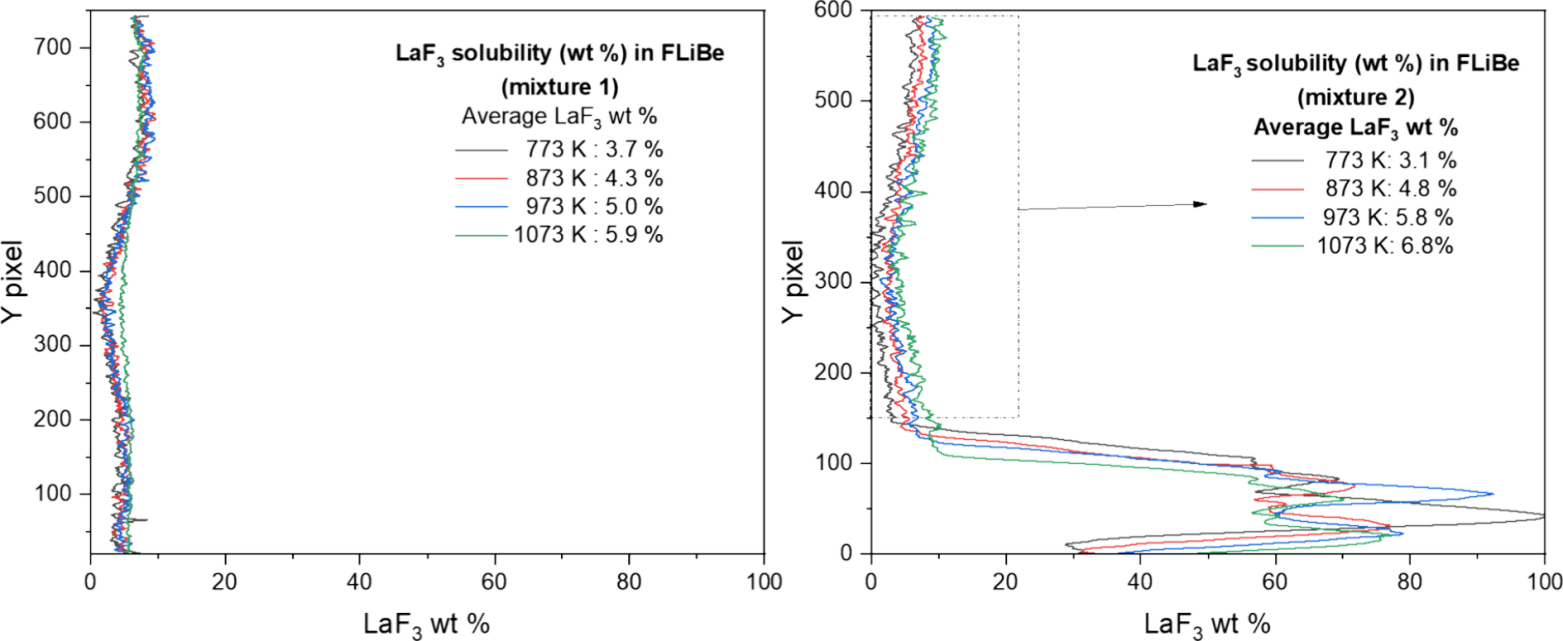
Comparison of LaF_3_ solubility measurements in mixtures of 10 wt % LaF_3_ in FLiBe (mixture 1) and 20 wt % LaF_3_ in FLiBe (mixture 2).

Results of the neutron attenuation determinations are given in Figure 6 for the LaF_3_–FLiBe mixtures. In this case, the solubility was observed to increase with temperature, from 1.8 ± 0.2 mol % at 773 K to 3.3 ± 0.2 mol % at 1,073 K.

The difference in solubility between the two systems was small, suggesting that the system with 20 wt % LaF_3_ (mixture 2) had reached saturation and the 10 wt % LaF_3_ (mixture 1) was very close to saturation. Comparison of the attenuation profiles of the homogeneous fluid region of the two salt systems (Figure 6) also suggested that the results were similar.

### 3.4 Density of LaF_3_–FLiBe Mixtures

**Figure 7:**
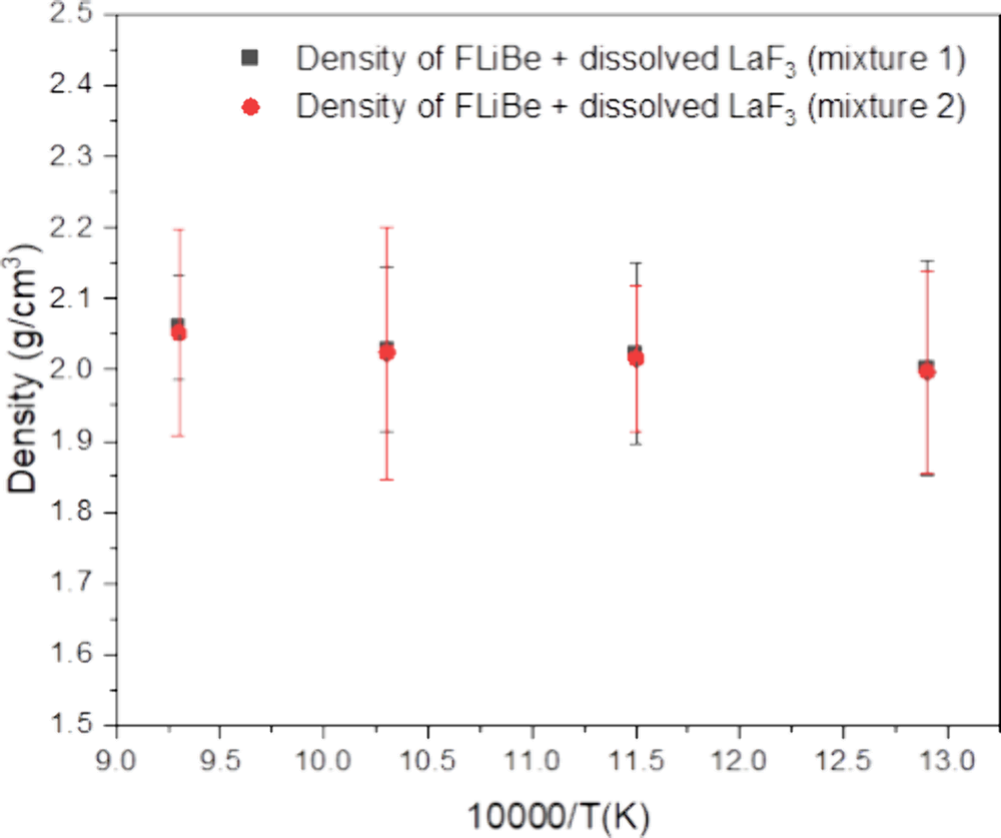
Density of LaF_3_–LiF–BeF_2_ system as a function of temperature for the mixtures measured in this study.

Once solubilities were determined, the densities of the LaF_3_–FLiBe mixtures were computed. These required literature data as given in the Supporting Information and assumptions about the mixing of the LaF_3_–LiF–BeF_2_ system. A Redlich–Kister model^32^ was used to calculate densities for LiF–BeF_2_ (Figure S3), and the interaction parameters are available.^33^ The resulting densities are given in eq 5. Details of the calculation and a plot of FLiBe density are provided in the Supporting Information. Interaction parameters are not available for the LaF_3_–LiF–BeF_2_ mixture. Hence, density correlations from Janz and Spedding were used for the LaF_3_ component of the mixture.^28,34^ An example is given for a calculation of 10 wt % LaF_3_ in FLiBe in the Supporting Information and Figure S4. The densities used in the analysis for the mixture are given in eq 6. In eq 4, the densities are in the units input into the equation and in g·cm^–3^ in eq 5. Temperature, *T*, is in kelvin.
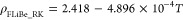
5
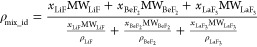
6For the system being studied, the solubility of LaF_3_ in the system varied with the temperature; therefore, the densities were recalculated for each system studied using the measured solubilities. These results are presented in Figure 7. The results from the two mixtures were very similar, suggesting that the solubility limit was reached throughout the temperature range studied in both systems. The density of the mixture changed little throughout the temperature range, dropping slightly with the temperature. The change was within the uncertainty of the measurement.

The resulting densities of the FLiBe + LaF_3_ mixtures are also given in Table 3 along with predictions by using the Redlich–Kister model. The densities of the mixtures between 773 and 973 K are consistently lower than those predicted by the Redlich–Kister model by 10%, with a generally flat profile being the same for both the experiment and the model. The deviation is higher at the highest temperature, 1,073 K. Thus, while both the experiment and the model predict an increase in density because of the higher concentration of LaF_3_, the measured increase is not as much as predicted by the model.

**Table 3:** Density Determinations for Two Mixtures of LaF_3_ in FLiBe between 773 K and 1,073 K

	Density of FLiBe + dissolved LaF_3_ (mixture 1)			Density of FLiBe + dissolved LaF_3_ (mixture 2)		
Temp (K)	LaF_3_ (mol fraction)	Experimental	Calculated	LaF_3_ (mol fraction)	Experimental	Calculated
773	0.77	2.00 ± 0.15	2.10	0.98	1.99 ± 0.14	2.11
873	1.68	2.02 ± 0.13	2.12	1.87	2.02 ± 0.11	2.13
973	2.20	2.03 ± 0.11	2.10	2.57	2.03 ± 0.18	2.13
1,073	4.13	2.06 ± 0.07	2.19	3.96	2.05 ± 0.14	2.18

## 4 Discussion

The solubilities of LaF_3_ were compared with calculations based on the Molten Salt Thermal Properties Database–Thermochemistry (MSTDB-TC).^35,36^ These calculations were based on the MSTDB-TC v.3 that includes the solubilities of PuF_3_, CeF_3_, and LaF_3_.^37^ Because the original data for LaF_3_ in FLiBe could not be found, the solubility of LaF_3_ in FLiBe was derived from optimized thermodynamic models after studying the subsystems of LiF-BeF_2_–ZrF_4_–UF_4_-LaF_3_ and correlations between CeF_3_ and PuF_3_. Relevant phase diagrams shown in Figures S5 and S6 were used to benchmark the database and ensure that the literature values^38–42^ could be reproduced in current calculations.

**Figure 8:**
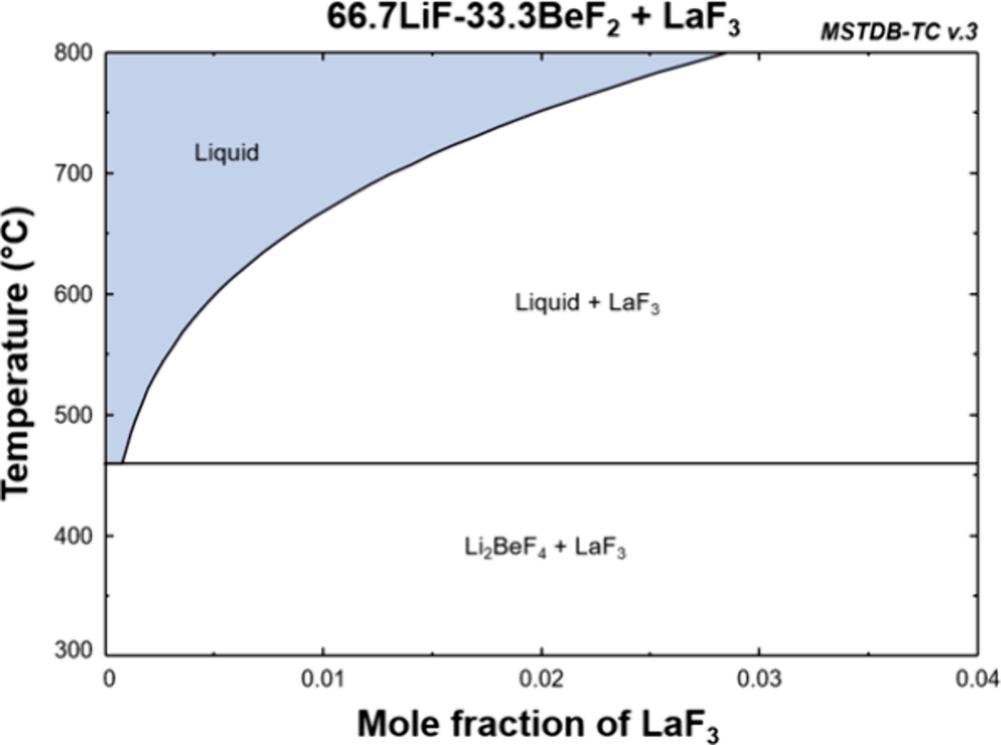
Pseudobinary phase diagram for LaF_3_ in FLiBe derived from MSTDB-TC v.3.

The literature systems included UF_4_, but Ward et al. showed that UF_4_ has a very small effect on the solubility of CeF_3_ (Figure S7).^38^ Assuming that a similarly negligible effect happens to LaF_3_ (Figure S6), the solubility of LaF_3_ could be derived from the computed phase diagram in Figure 8 for FLiBe + LaF_3_.

From the phase diagram shown in Figure 8, the solubility of LaF_3_ was calculated, as shown in Table 4.

**Table 4:** Computed Solubility of LaF_3_ in FLiBe

Temp (K)	Solubility in 66.7LiF-33.3BeF_2_ (mol %)	Average experimental value (mol %)
**773**	**0.155**	**0.88 ± 0.17**
798	0.218	
823	0.300	
848	0.405	
**873**	**0.536**	**1.78 ± 0.08**
898	0.698	
923	0.896	
948	1.136	
**973**	**1.424**	**2.38 ± 0.64**
998	1.769	
1,023	2.180	
1,048	2.672	
**1,073**	**3.262**	**4.04 ± 0.37**

**Figure 9:**
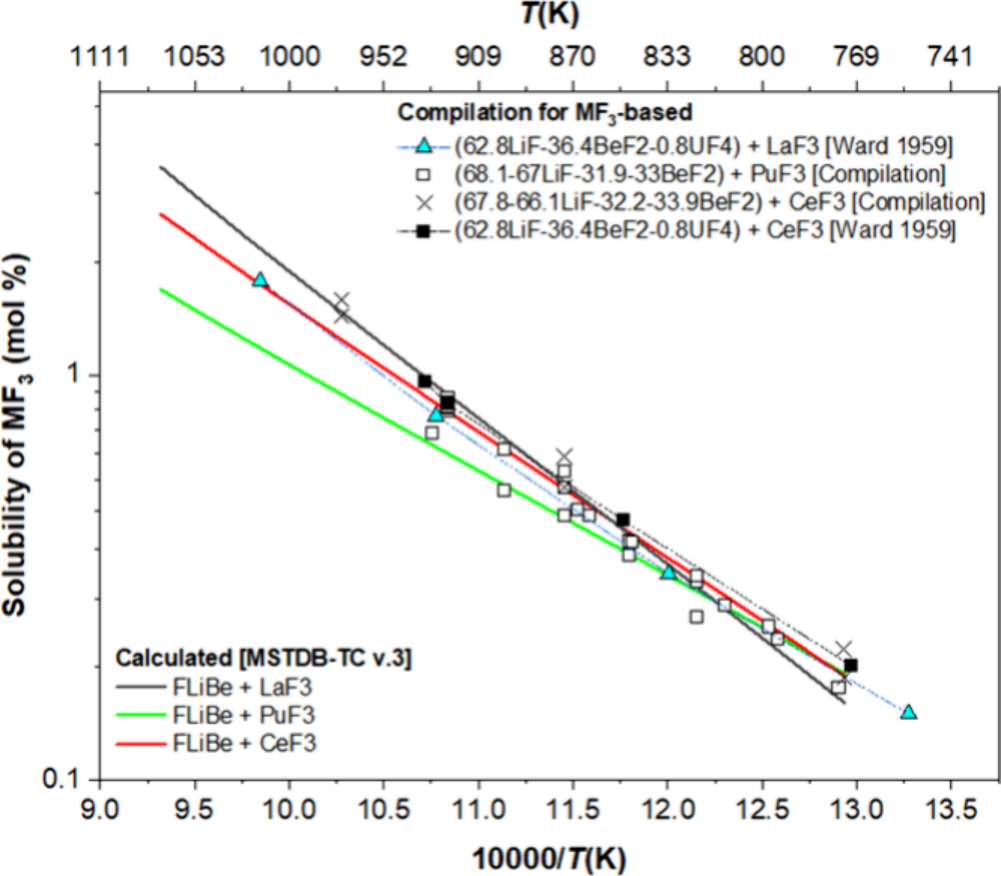
Comparison of MSTDB-TC v.3 calculations and published literature of PuF_3_, CeF_3_, and LaF_3_ solubility measurements.

**Figure 10:**
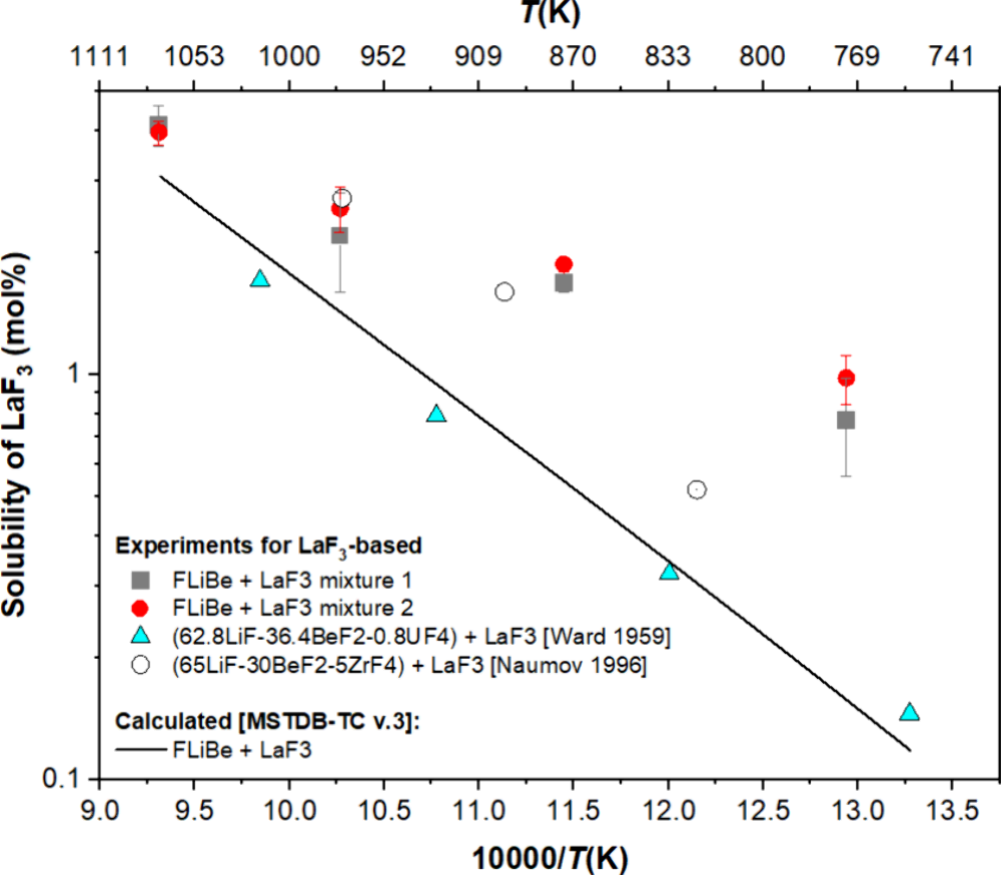
Comparison of MSTDB-TC v.3 calculations and published literature with LaF_3_ solubility measurements.

The computed values were plotted showing little difference in the solubility expected among CeF_3_, PuF_3_, and LaF_3_ (Figure 9). The values show that these salts overlap closely in the temperature range studied. The experimental results were plotted on the same axes (Figure 10) with models and experimental results taken from earlier publications. The experimental data show that there is good overlap with published results throughout the temperature range; however, the experimental data show that there may be some nonlinearity in the plot that is not reflected in the model. The deviation from the linear slope may arise from the supersaturation of LaF_3_ in the LiF–BeF_2_ system.

When comparing the results of the solubility measurements with earlier literature^16,41^ and calculated values, the system may have become slightly supersaturated at the highest temperature achieved in the experiment (i.e., 1,073 K), which translates to supersaturation throughout the rest of the experimental program over subsequent heating and cooling cycles.

## 5 Conclusions

Thermophysical properties of fluoride salts were measured by neutron radiography, including a FLiBe eutectic and mixtures of FLiBe with additions of 10 wt % LaF_3_ and 20 wt % LaF_3_, respectively. The density of FLiBe was measured from 773 to 1,073 K by dilatometry and by neutron radiography. The measurements involved cycling of the salt above the melting point at least twice to check for hysteresis, removal of bubbles and voids, and changes in composition over 24 h. The density data for FLiBe agree with published data and indicate that both the dilatometry measurements and the neutron attenuation data can be used to determine the density.

The solubility of LaF_3_ in FLiBe was measured in two systems: one that indicated from a precipitate in contact with the salt that the solution was saturated at all the temperatures investigated, 20 wt % LaF_3_ added to FliBe (mixture 2), and one from a 10 wt % system (mixture 1) in which salt crystals became lodged above the molten FLiBe. The results showed that the LaF_3_ uptake into the salt reached a value close to that predicted by phase diagrams generated by the MSTDB-TC v.3 at 1,073 K, up to 4 mol %. However, curvature in the salt solubility plot, or higher than predicted solubility observed at lower temperatures, suggested evidence of supersaturation of LaF_3_ in the FLiBe system, because of thermal cycling of the salt.

## Supporting Information Available

Neutron radiography data, calculation of FLiBe density from volume, calculation of mixture densities, and calculation of phase diagrams for the mixture of LiF-BeF_2_ and LaF_3_, (PDF)
