# Supplementary material for: Density Measurements of Molten LiF–BeF2 and LiF–BeF2–LaF3 Salt Mixtures by Neutron Radiography
Source: ACS Omega. 2024 Jun 10;9(25):27204–13. doi: 10.1021/acsomega.4c01446 (PMC11209898; doi:10.1021/acsomega.4c01446)
Supplement: Supplementary file 1 — ao4c01446_si_001.pdf [file ao4c01446_si_001.pdf]

Supplemental Information

Density Measurements of Molten LiF-BeF_2 and LiF-BeF_2-LaF_3 Salt Mixtures by Neutron Radiography

Jisue Moon[1], Joanna McFarlane[*1], Hunter B. Andrews[1], Kevin R. Robb[1], Molly Ross[1], Dino Sulejmanovic[1], Yuxuan Zhang[1], Erik Stringfellow[1], Can Agca[1], Juliano Schorne-Pinto[2], Theodore M. Besmann[2],

1. Oak Ridge National Laboratory, Oak Ridge, TN
2. University of South Carolina, Columbia, SC

[*1] Corresponding author, mcfarlanej@ornl.gov, Oak Ridge National Laboratory, PO Box 2008 Oak Ridge TN, 37831.

Notice of copyright
This manuscript has been authored by UT-Battelle LLC under contract DE-AC05-00OR22725 with the US Department of Energy (DOE). The US government retains and the publisher, by accepting the article for publication, acknowledges that the US government retains a nonexclusive, paid-up, irrevocable, worldwide license to publish or reproduce the published form of this manuscript, or allow others to do so, for US government purposes. DOE will provide public access to these results of federally sponsored research in accordance with the DOE Public Access Plan (http://energy.gov/downloads/ doe-public-access-plan).

SI-1. Neutron Radiography Data

Figure S1 shows a selection of neutron radiography images taken during the heating and cooling processes. Measurements were collected in increments of 100 K from 773 K to 1,073 K for the heating process, shown in Figures S1 (a)–S1(d), and from 1,073 K to 773 K for the cooling process, shown in Figures S1 (e)–S1 (g). The samples were loaded in FLiBe with 20 wt % LaF_3 or mixture 2 (left) and 10 wt % LaF_3 or mixture 1 (right), and neat FLiBe was measured as a reference (middle). Figure 3 shows the transmission profile at each temperature from Figure S1, with solid and dotted lines indicating the heating and cooling processes, respectively.

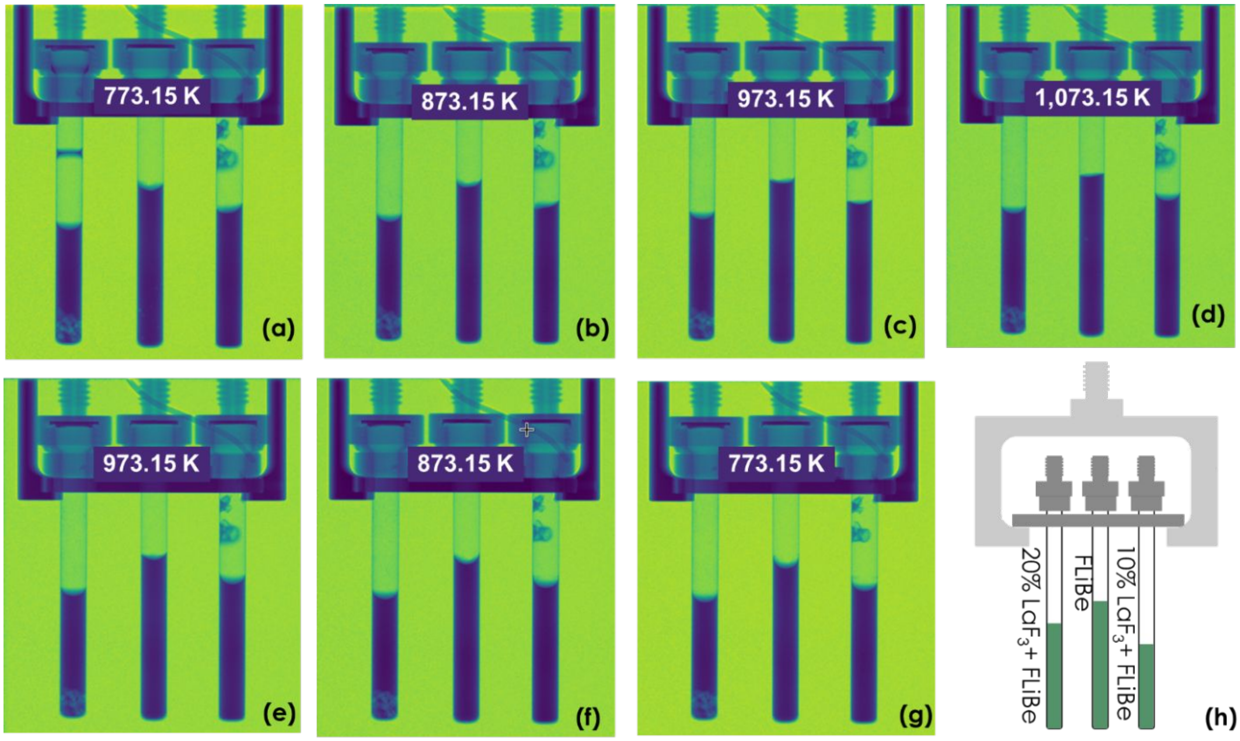

Figure S1. Transmission images of 20% LaF_3 + FLiBe or mixture 2 (left tube), FLiBe (middle tube), and 10% LaF_3 + FLiBe or mixture 1 (right tube). Images (a), (b), (c), and (d) were collected as the temperature increased, and images (e),(f), and (g) were measured as the temperature decreased. Image (h) shows an overview of the collected measurements.

The neutron radiographs for the FLiBe sample only showed subtle changes during the temperature ramp from 773 K to 1,073 K. Radiographs taken at each temperature are shown in Figure S2.

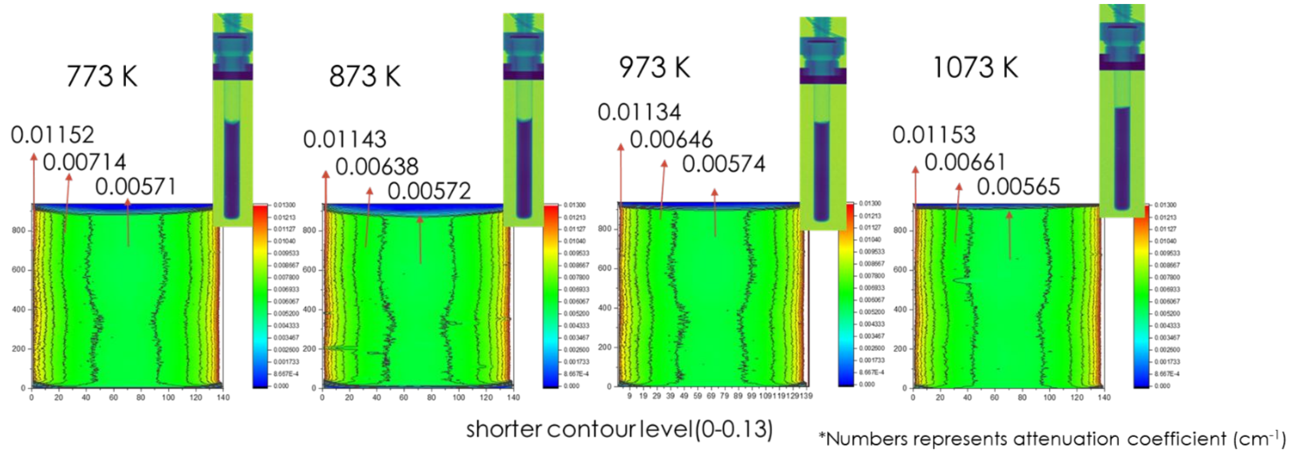

Figure S2. The mapping of neutron attenuation on the radiographs of the FLiBe sample for the four hold temperatures spaced 100 K apart.

SI-2. Calculation of FLiBe Density from Volume

To calculate the total volume of the FLiBe, the FLiBe is divided into three regions. Region 1 is the region from the top of the meniscus to the bottom of the meniscus. Region 2 is the region from the bottom of the meniscus to the top of the curved section at the bottom of the V can. Region 3 is the curved portion at the bottom of the can. Each of these regions is defined by the height of the region, h, measured in pixels, and the pixel diameter of the V can, d_p . The total volume, in pixels^3, is then defined by

$$V_{total} = \pi d_p^2\left(\frac{h_1}{12} + \frac{h_2}{4} + \frac{h_3}{6}\right)$$

To convert the volume from pixels^3 to cm^3, the inner diameter of the can was used as a reference. The inner diameter, d_0, of several vanadium cans were measured at 20°C. The diameter of the cans at temperature, T, can then be calculated from the coefficient of thermal expansion by

$$d_{cm} = d_0(1 + \alpha(T - T_0)$$

The value of α, in K^{-1}, as a function of T, in units of K, is given by the following relation [27]

$$\alpha = 1.646e - 15T^3 - 2.551E - 12T^2 + 2.277E - 9T - 6.129E - 4T^{-1}$$

The total volume, in cm, is then defined by

$$V_{total} = \pi\left(\frac{h_1}{12} + \frac{h_2}{4} + \frac{h_3}{6}\right)\frac{d_{cm}^3}{d_p}$$

The density is then the mass divided by volume and can be written as

$$\rho = \frac{m_{FLiBe} d_p}{\pi(d_0(1 + \alpha(T - T_0)))^3\left(\frac{h_1}{12} + \frac{h_2}{4} + \frac{h_3}{6}\right)}$$

The overall error of the density calculation is defined by the standard error propagation formula for N number of variables, v, each with some error, σ.

$$\sigma_\rho = \sqrt{\sum_{n=1}^{N} \left(\frac{\partial \rho}{\partial v_n}\right)^2 \sigma_{v_n}^2}$$

Each of the derivative terms in the error propagation formula is given by

$$\frac{\partial \rho}{\partial m} = \frac{d_p}{\pi(d_0(1+\alpha(T-T_0)))^3\left(\frac{h_1}{12}+\frac{h_2}{4}+\frac{h_3}{6}\right)}$$

$$\frac{\partial \rho}{\partial d_p} = \frac{m}{\pi(d_0(1+\alpha(T-T_0)))^3\left(\frac{h_1}{12}+\frac{h_2}{4}+\frac{h_3}{6}\right)}$$

$$\frac{\partial \rho}{\partial d_0} = \frac{-3d_p m\alpha(T-T_0)}{\pi(d_0(1+\alpha(T-T_0)))^4\left(\frac{h_1}{12}+\frac{h_2}{4}+\frac{h_3}{6}\right)}$$

$$\frac{\partial \rho}{\partial \alpha} = \frac{-3d_p m d_0(T-T_0)}{\pi(d_0(1+\alpha(T-T_0)))^4\left(\frac{h_1}{12}+\frac{h_2}{4}+\frac{h_3}{6}\right)}$$

$$\frac{\partial \rho}{\partial T} = \frac{-3d_p m d_0\alpha}{\pi(d_0(1+\alpha(T-T_0)))^4\left(\frac{h_1}{12}+\frac{h_2}{4}+\frac{h_3}{6}\right)}$$

$$\frac{\partial \rho}{\partial T_0} = \frac{3d_p m d_0\alpha}{\pi(d_0(1+\alpha(T-T_0)))^4\left(\frac{h_1}{12}+\frac{h_2}{4}+\frac{h_3}{6}\right)}$$

$$\frac{\partial \rho}{\partial h_1} = \frac{m d_p}{12\pi(d_0(1+\alpha(T-T_0)))^3\left(\frac{h_1}{12}+\frac{h_2}{4}+\frac{h_3}{6}\right)^2}$$

$$\frac{\partial \rho}{\partial h_2} = \frac{m d_p}{4\pi(d_0(1+\alpha(T-T_0)))^3\left(\frac{h_1}{12}+\frac{h_2}{4}+\frac{h_3}{6}\right)^2}$$

$$\frac{\partial \rho}{\partial h_3} = \frac{m d_p}{6\pi(d_0(1+\alpha(T-T_0)))^3\left(\frac{h_1}{12}+\frac{h_2}{4}+\frac{h_3}{6}\right)^2}$$

The measurement or calculation error for each of the variables is shown in Table S1.

Table S1: Density calculation variable error values

Variable	Definition	Error (σ)
m	Mass FLiBe	0.005 g
d_p	Diameter of vanadium can, measured in pixels	5 Pixels
d_0	Diameter of vanadium can in cm measured at 20°C	3.86e-4 cm

α	Vanadium coefficient of thermal expansion	0.01α K^{-1} [27]
T	Temperature of FLiBe density measurement	0.5°C
T_0	Temperature of vanadium can reference measurement (20°C)	0.5°C
h_1	Height of region 1 (meniscus)	5 pixels
h_2	Height of region 2 (primary cylinder)	5 pixels
h_3	Height of region 3 (bottom of the can)	5 pixels

SI-3. Calculation of Mixture Densities

The densities FLiBe and FLiBe + LaF_3 were calculated by arithmetic mean or by the Redlich–Kister model. The end point densities were taken from the Janz compilation for fluoride salts [29]. These included the endpoint members LiF, BeF_2, and LaF_3. Density of LaF_3-LiF pseudobinary mixtures are compiled from Janz [28] in Table 1. Figure S3 shows FLiBe density comparison with Cantor's experimental data [39, 40] and ideal/RK models calculated by Birri et al. [33].

Table S2: LaF_3-LiF experimental densities taken from Janz [28]

LaF_3 mol%	Density Equation	Temperature Range (K)
0.0	d = 2.074 – 3.321 x 10^{-4} T	1130-1350
5.0	d = 2.491 – 3.648 x 10^{-4} T	1140-1350
10.0	d = 2.799 – 3.118 x 10^{-4} T	1180-1350
15.0	d = 3.500 – 6.658 x 10^{-4} T	1110-1350
20.0	d = 3.737 – 6.486 x 10^{-4} T	1130-1350
25.0	d = 3.799 – 5.507 x 10^{-4} T	1130-1350
100	d = 5.793 – 6.82 x 10^{-4} T	1750-2450

Using values in Table 1, we performed RK modeling on LaF_3-LiF molten salt pseudobinary. Both the ideal behavior and the RK modeled densities lie within 2% of the Table S1 experimental data. However, is is apparent that the values for pure LiF in Table S1 are not in good agreement with those reported by Cantor [39]. Both equations apply to higher temperatures than measured in this work.

LiF:

d = 2.370575 – 5.0 × 10^{-4}*T | 1,124 K–1,368 K | <1% | [39]

BeF_2:

d = 1.972 – 1.45 × 10^{-5}*T | 1,073 K–1,123 K | ±0.5% | [40]

FLiBe mixture, 1,073 K–1368 K:

$$\boldsymbol{\rho_{LiF}} = 2.370575 - 5.0 \times 10^{-4} * \mathrm{T}\ ,$$

$$\boldsymbol{\rho_{BeF_2}} = 1.972 - 1.45 \times 10^{-5} * \mathrm{T}\ ,$$

$$\boldsymbol{\rho_{FLiBe\_id}} = \frac{x_{LiF}MW_{LiF} + x_{BeF_2}MW_{BeF_2}}{\frac{x_{LiF}MW_{LiF}}{\rho_{LiF}} + \frac{x_{BeF_2}MW_{BeF_2}}{\rho_{BeF_2}}},$$

$$\rho_{FLiBe\_id} = \frac{0.66 * 25.939 + 0.34 * 47.01}{\frac{0.66 * 25.939}{2.370575 - 5.0 \times 10^{-4} * \mathrm{T}} + \frac{0.34 * 47.01}{1.972 - 1.45 \times 10^{-5} * \mathrm{T}}},$$

$$\rho_{FLiBe\_nid} = \rho_{FLiBe\_id} + \rho_{FLiBe\_ex},$$

$$\rho_{FLiBe\_ex} = x_{LiF}x_{BeF_2}[A_1 + B_1 * T + (A_2 + B_2 * T) * (x_{LiF} - x_{BeF_2})],$$

$$\boldsymbol{A_1} = 0.893 \mid \boldsymbol{B_1} = -8.96 * 10^{-4} \mid \boldsymbol{A_2} = 0.378 \mid \boldsymbol{B_2} = -1.16 * 10^{-4},$$

$$\rho_{FLiBe} = \rho_{FLiBe\_id} + \rho_{FLiBe\_ex}.$$

Linearized results:

$$\rho_{FLiBe\_RK} = 2.418 - 4.896 \times 10^{-4} * T,$$

$$\rho_{FLiBe\_ideal} = 2.191 - 2.802 \times 10^{-4} * T.$$

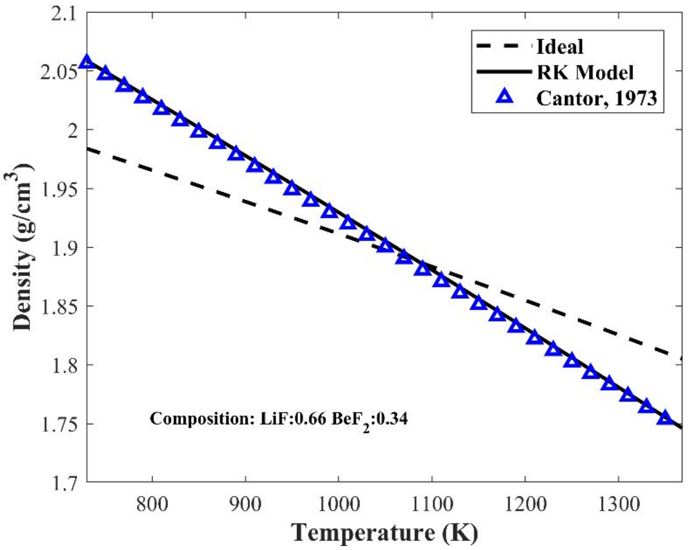

Figure S3. FLiBe density calculated with parameters from Birri et al. [33].

FLiBe + 10 wt % LaF_3 (1.84 mol %) mixture:

$$\rho_{mix} = \rho_{mix\_id} + \rho_{mix\_ex},$$

$$\rho_{LaF_3} = 5.793 - 6.82 \times 10^{-4} * \mathrm{T},$$

$$\rho_{mix\_id} = \frac{x_{LiF}MW_{LiF} + x_{BeF_2}MW_{BeF_2} + x_{LaF_3}MW_{LaF_3}}{\frac{x_{LiF}MW_{LiF}}{\rho_{LiF}} + \frac{x_{BeF_2}MW_{BeF_2}}{\rho_{BeF_2}} + \frac{x_{LaF_3}MW_{LaF_3}}{\rho_{LaF_3}}},$$

$$\rho_{mix\_ex} = x_{LiF}x_{BeF_2}[A_1 + B_1 * T + (A_2 + B_2 * T) * (x_{LiF} - x_{BeF_2})] + x_{LiF}x_{LaF_3}(A_3 + B_3 * T)$$

$$\rho_{mix\_id} = \frac{0.6478*25.939+0.3337*47.01+0.0184*195.9}{\frac{0.6478*25.939}{2.370575 - 5.0 \times 10^{-4}*T} + \frac{0.3337*47.01}{1.972 - 1.45 \times 10^{-5}*T} + \frac{0.0184*195.9}{5.793 - 6.82 \times 10^{-4}*T}},$$

Interaction parameters with LaF_3-BeF_2 were not given. LaF_3-LiF system is RK modeled using the experimental data in Table S1. It turned out to be a first order system with parameters below.

$$\boldsymbol{A_3} = 0.361 \mid \boldsymbol{B_3} = -3.73 * 10^{-4} \mid$$

The linearized result is shown below and plotted in Figure S4.

Linearized result, assuming 1.84 mol % LaF_3 in FLiBe:

$$\boldsymbol{\rho_{mix\_RK} = 2.559 - 5.038 \times 10^{-4} * T},$$

$$\boldsymbol{\rho_{mix\_ideal} = 2.336 - 2.978 \times 10^{-4} * T}.$$

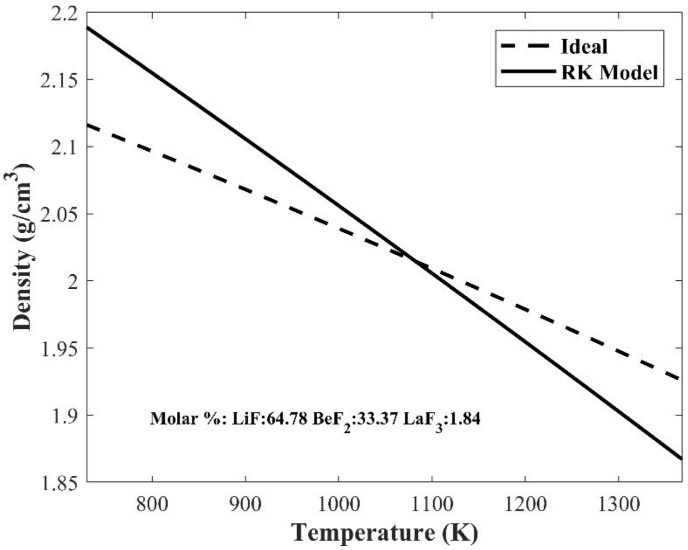

Figure S4. Calculated densities of FLiBe + 10 wt % LaF_3 (1.84 mol %).

SI-4. Calculation of Phase Diagrams for the Mixture of LiF-BeF_2 and LaF_3

Binary phase diagrams were used to benchmark the approach in determining the pseudoternary phase diagram. The solubility of LaF_3 in 65LiF-30BeF_2-5ZrF_4 computed using MSTDB-TC v.3 vs. experimental values from Naumov et al. [18] is shown in Figure S5. The solubility of LaF_3 in 62.8LiF-36.4BeF_2-0.8UF_4

computed using MSTDB-TC v.3 vs. experimental values from Ward et al. is shown in Figure S6. The effect of UF_3 on the solubility of CeF_3 in FLiBe is shown in Figure S7.

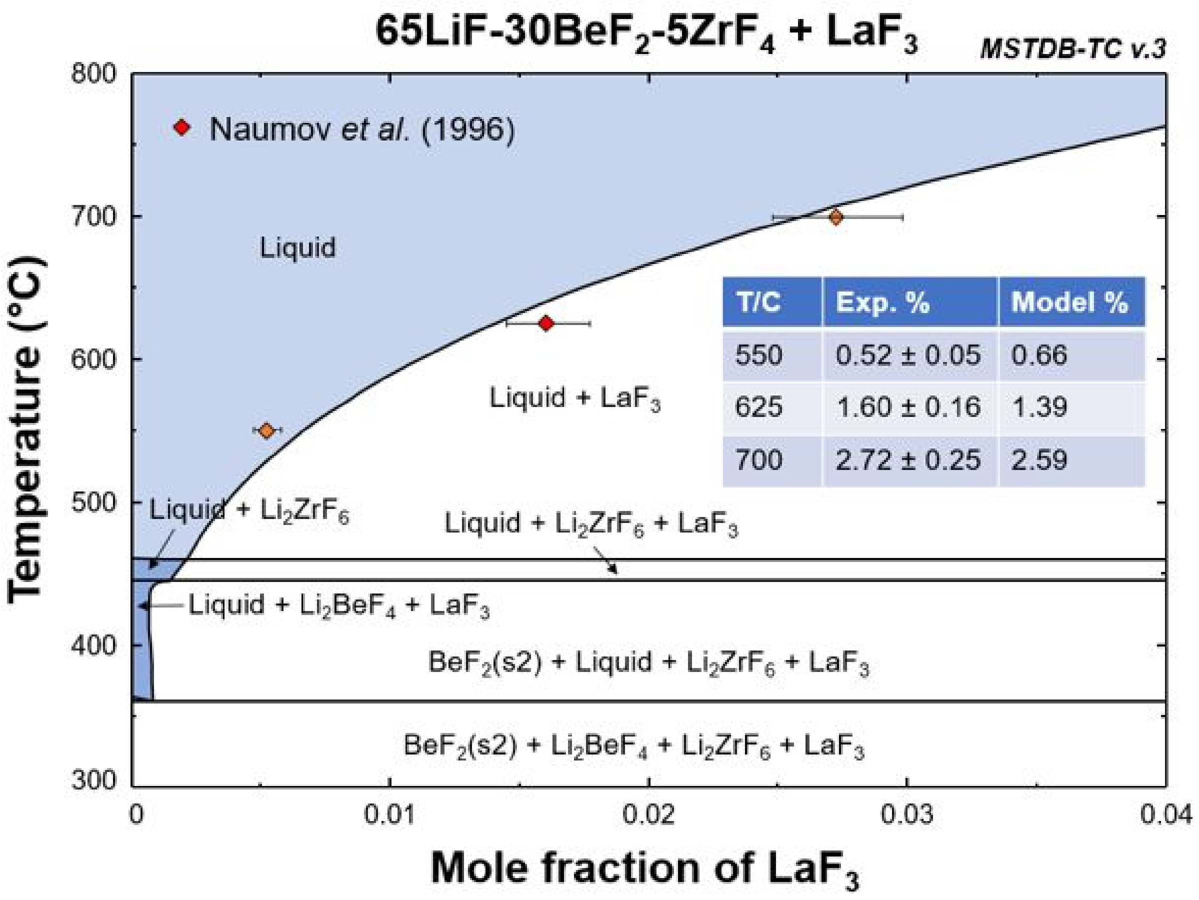

Figure S5. Solubility of LaF_3 in 65LiF-30BeF_2-5ZrF_4 computed using MSTDB-TC v.3 vs. experimental values from Naumov et al. [41]. These data are also accessible from Lizin and Tomilin [42].

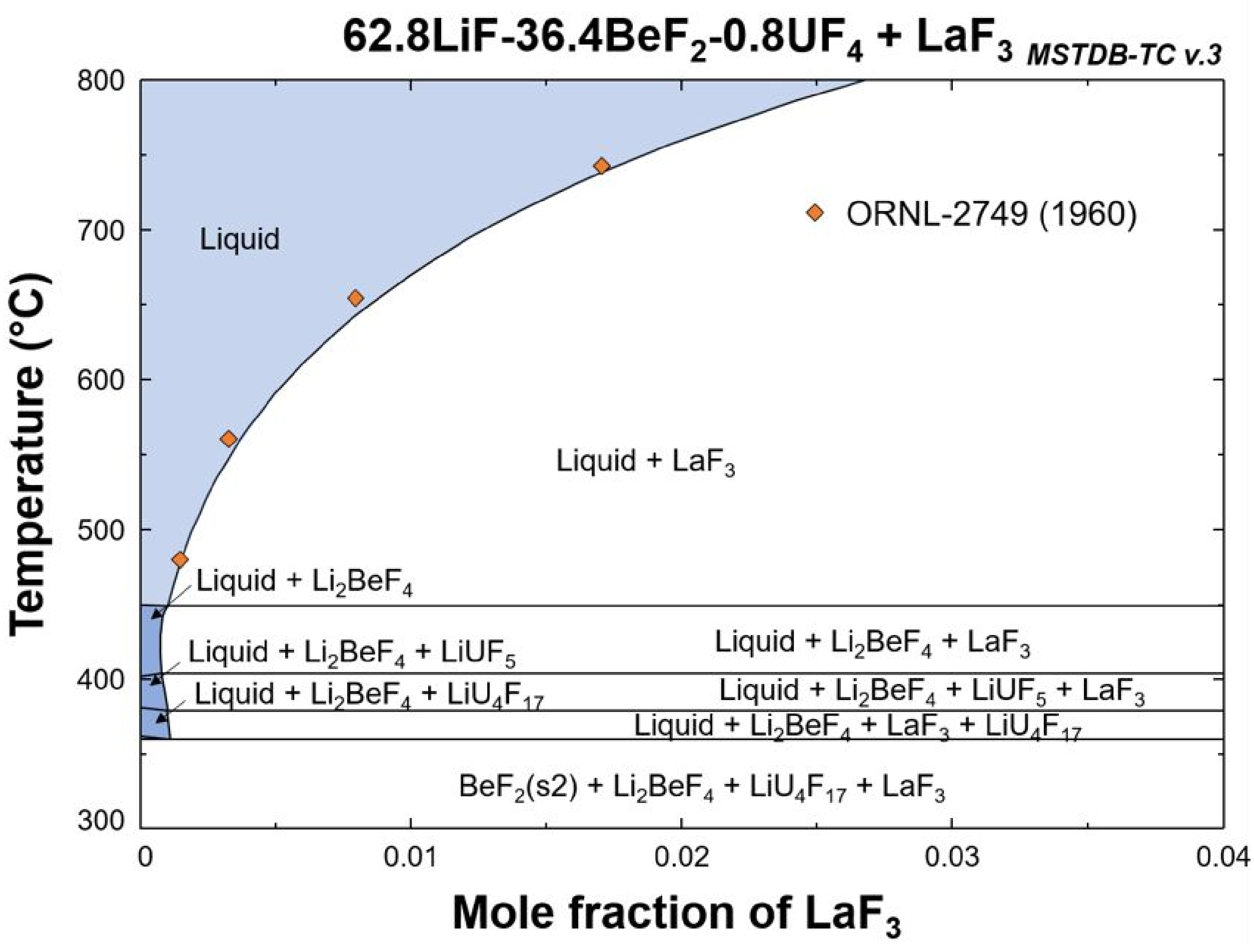

Figure S6. Solubility of LaF_3 in 62.8LiF-36.4BeF_2-0.8UF_4 computed using MSTDB-TC v.3 vs. experimental values from Ward et al. [38].

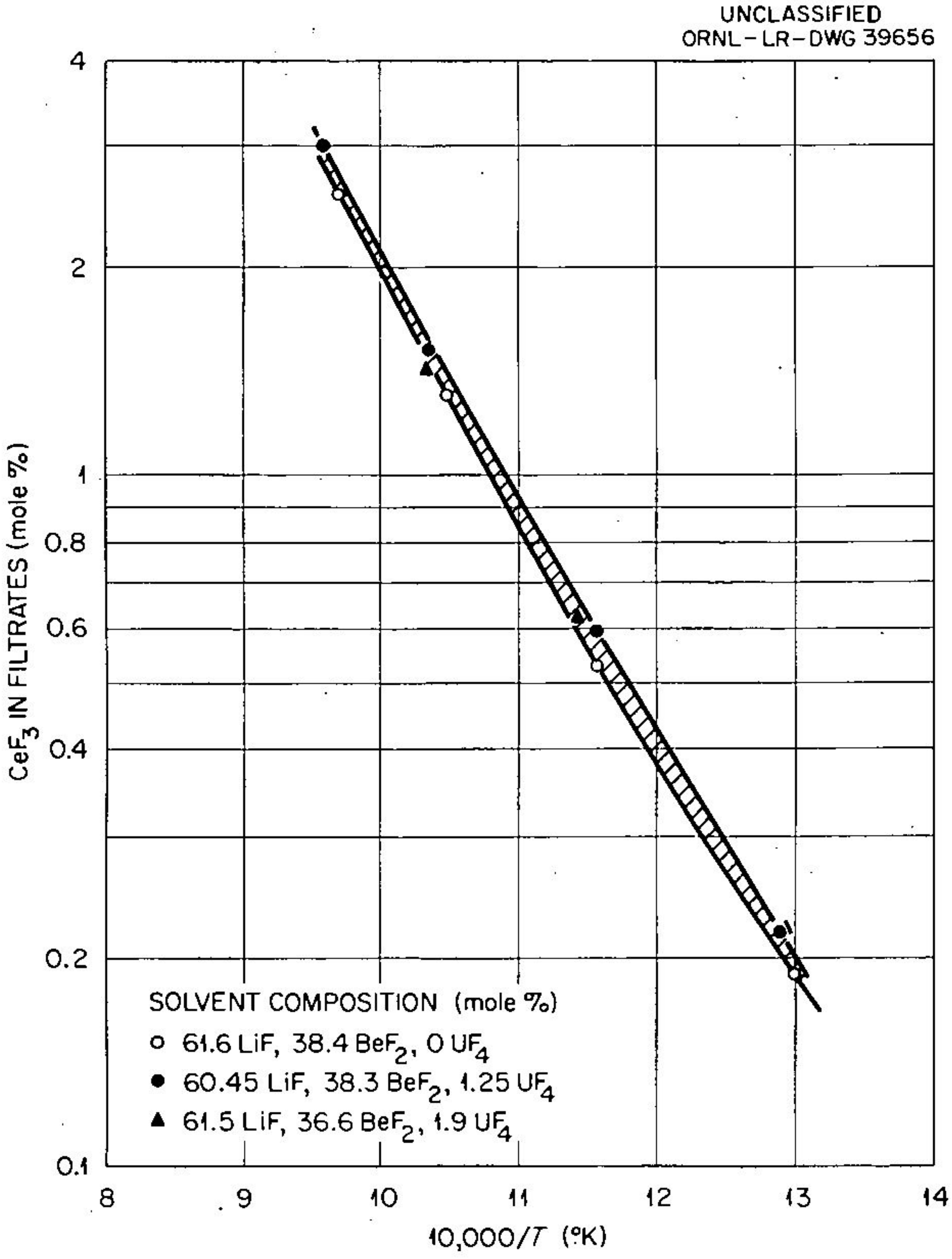

Figure S7. Effect of UF_4 on the solubility of CeF_3 in LiF-BeF_2 (62-38 mol %) as reproduced from Ward et al. 1965 [38]. The solvent composition was calculated from average values of filtrate analyses.
